# Time to revisit the problem of CIN? The low incidence of acute kidney injury with and without contrast in hospitalized patients: an observational cohort study

**DOI:** 10.1186/s40697-015-0073-6

**Published:** 2015-10-12

**Authors:** Juliya Hemmett, Lee Er, Helen H. L. Chiu, Christopher Cheung, Ognjenka Djurdjev, Adeera Levin

**Affiliations:** Division of Nephrology, University of Western Ontario, London, ON Canada; BC Provincial Renal Agency, Vancouver, BC Canada; Division of Nephrology, Department of Medicine, Faculty of Medicine, University of British Columbia, 6010A, 1081 Burrard Street, Vancouver, BC V6Z 1Y6 Canada

## Abstract

**Background:**

Acute kidney injury (AKI) following imaging procedures with contrast medium in hospitalized patients is commonly attributed to contrast-induced nephropathy (CIN). This study sought to establish a benchmark of the incidence of AKI in hospitalized patients who underwent computed tomography (CT) scans, with and without intravenous contrast administration.

**Methods:**

This was a multi-center observational cohort study. Hospitalized patients in four hospitals with CT scans during two time periods in 2012 and 2013 were included. AKI post-scan was defined as a change in serum creatinine (sCr) in absolute terms of ≥26.5 μmol/L (≥0.3 mg/dl), occurring within 7 days of the CT scan. AKI incidence was examined by study phases and CT-scan types using logistic regression models. Multinomial logistic regression was used to examine the proportions of sCr availability between two study phases.

**Results:**

Three hundred and twenty-five patients in Period 1 and 518 patients in Period 2 were included in the study. The incidence of AKI in Period 1 was similar in those who received contrast and in those who did not (11.6 % [95 % C.I.: 6.5, 18.7] vs. 10.1 % [95 % C.I.: 5.1, 17.3]; *p* = 0.38). The incidence of AKI remained not significantly different between the two periods in those who received contrast (11.6 % [95 % C.I.: 6.5, 18.7] vs. 10.7 % [95 % C.I.: 6.8, 15.8]; *p* = 0.89) and those who did not (10.1 % [95 % C.I.: 5.1, 17.3] vs. 9.1 % [95 % C.I.: 5.2, 14.6]; *p* = 0.54). Among those who received contrast, there was a significant increase in the availability of both pre- and post- CT scan sCr in Period 2 compared to Period 1 (73.6 % [95 % C.I.: 67.7, 80.6] vs. 79.8 % [95 % C.I.: 75.2, 84.7]; *p* = 0.006).

**Limitations:**

Our study was not targeted to specifically assess the impact of a prevention protocol on the incidence of AKI and was limited to settings within one health authority in the province.

**Conclusion:**

In hospitalized patients, the incidence of AKI is low, not different between those who did and did not receive contrast, and was not impacted by improvement in the monitoring of sCr in at risk patients. A better understanding of the determinants of AKI post-contrast scan is required to improve strategies to reduce the incidence of AKI.

## Abrégé

**Données connues:**

L’occurrence d’un épisode d’insuffisance rénale aiguë (IRA) à la suite d’examens d’imagerie médicale avec administration d’un agent de contraste est fréquemment attribuée à une néphropathie induite par l’agent de contraste lui-même. La présente étude a cherché à établir des bases de référence susceptibles d’aider à mesurer l’incidence des épisodes d’IRA chez les patients hospitalisés qui ont à subir un examen par tomodensitométrie (scanner), avec ou sans administration intraveineuse d’un agent de contraste.

**Méthodologie:**

Cette étude observationnelle a été réalisée sur des cohortes de patients hospitalisés sélectionnés dans les unités de néphrologie de quatre centres hospitaliers différents. Ces patients ont subi des examens par tomodensitométrie au cours de deux périodes distinctes en 2012 et en 2013. Il a été décrété que les patients étaient atteints de néphropathie post-scanner lorsque leur taux de créatinine sérique augmentait de plus de 26.5 μmol/L ou à 0.3 mg/dl dans les 7 jours suivant l’examen. L’incidence d’insuffisance rénale aiguë a été analysée à l’aide d’un modèle de régression logistique en fonction de la phase de l’étude et du type de tomodensitomètre utilisé pour l’examen. Une régression logistique multinomiale a été utilisée pour présenter les taux de créatinine sérique mesurés entre les phases de l’étude.

**Résultats:**

La cohorte de la phase 1 comptait 325 patients et celle de la phase 2 en comptait 518. Il en est ressorti que l’incidence d’IRA post-scanner était similaire chez tous les patients de la phase 1, qu’ils aient ou non reçu un agent de contraste par intraveineuse avant l’examen (11.6 % [95 % I.C: 6.5, 18.7] vs 10.1 % [95 % I.C: 5.1, 17.3]; *p* = 0.38). L’incidence des épisodes d’IRA post-scanner est demeurée similaire dans les deux phases pour les patients ayant reçu un agent de contraste (11.6 % [95 % I.C: 6.5, 18.7] vs 10.7 % [95 % I.C: 6.8, 15.8]; *p* = 0.89) de même pour les patients n’en ayant pas reçu (10.1 % [95 % I.C: 5.1, 17.3] vs 9.1 % [95 % I.C: 5.2, 14.6]; *p* = 0.54). Chez les sujets ayant reçu un produit de contraste, il y avait une plus grande disponibilité des mesures de créatinine sérique pré — et post-scanner dans la période 2 par rapport à la période 1 (73.6 % [95 % I.C: 67.7, 80.6] vs 79.8 % [95 % I.C: 75.2, 84.7]; *p* = 0.006).

**Limites de l’étude:**

La présente étude ne visait pas à évaluer de façon systématique l’impact d’un protocole de prévention sur l’incidence d’un épisode d’IRA, étant donné qu’elle s’est limitée aux paramètres établis dans un seul cadre régional.

**Conclusion:**

L’incidence d’un épisode d’IRA chez les patients hospitalisés subissant un examen en tomodensitométrie demeure faible, et n’apparait pas significativement différente qu’ils reçoivent ou non un agent de contraste au préalable. Une surveillance plus rigoureuse des taux de créatinine sérique chez les patients à risque n’a pas non plus mené à des différences marquées dans l’incidence d’un épisode d’IRA post-scanner. Ainsi, une meilleure compréhension des facteurs susceptibles de provoquer des épisodes d’IRA post-scanner est requise afin d’améliorer les stratégies visant la réduction de leur incidence.

## What was known before

Contrast induced nephropathy (CIN) is a term used to describe AKI after the receipt of contrast needed for imaging studies. It is well described in specific populations undergoing contrast dye studies, but the true incidence is not well known.

## What this study adds

Before we can appropriately address the perceived risk of AKI attributed to the administration of contrast medium for diagnostic scans, the incidence of AKI in hospitalized patients subsequent to contrast CT scans should be explored. In this study, we found that the incidence of AKI was low in hospitalized patients who received CT scans, and was not different between patients who received non-contrast and IV contrast scans. Prevention strategies targeting AKI in general as opposed to specifically contrast-induced AKI in hospitalized patients warrant further investigation.

## Background

Acute kidney injury (AKI) is a prevalent syndrome that is independently associated with high morbidity and mortality [1–4]. The current KDIGO guidelines define AKI according to both changes in serum creatinine (sCr) and urine output: changes in sCr as ≥26.5 μmol/L (≥0.3 mg/dl) constitute at least stage 1 AKI [5]. These small changes confer large risk, as demonstrated in large data sets [2, 6]. Notably, AKI is due to diverse etiologies [5].

One of the etiologies of AKI, contrast-induced nephropathy (CIN) or contrast-induced AKI, is an iatrogenic condition resulting from the administration of iodinated contrast medium used to enhance diagnostic accuracy of radiological examinations. It has traditionally been defined as a relative increase from baseline sCr values of >25 % or an absolute rise in sCr of >44 μmol/L (0.5 mg/dl), 48–72 h after intravascular administration of contrast medium, without an alternative cause of AKI [5, 7–9]. This is a broader definition than the newer KDIGO AKI definition [5]. Irrespective of the specific change in sCr used to define AKI, it is challenging to determine whether or not CIN can truly be attributed to contrast toxicity alone given the complexity of the medical conditions that patients undergoing contrast studies have [10, 11]. The incidence of CIN in the literature has ranged from 10 to 30 %, in part due to variability in definitions, and populations studied. Multiple reports describe the importance of small changes in sCr or AKI by conventional definitions, as being consistently associated with a higher risk of long-term mortality and dialysis, regardless of etiology [11–13]. Therefore, it would behoove us to understand the incidence of AKI, in the context of contrast administration, according to these new definitions. Given that contrast-induced AKI, or CIN, is perceived as an important modifiable cause of AKI in hospitalized patients, numerous research and quality improvement attempts have been made to prevent AKI post-administration of contrast medium for patient safety [13, 14].

Our multi-institutional study took advantage of the data collected in a recent quality improvement initiative in a regional health authority to establish a benchmark of the incidence of AKI in hospitalized patients who underwent computed tomography (CT) scans, with and without administration of intravenous (IV) contrast medium. We further examined the impact of the quality improvement strategy on ascertainment of renal function before and after the CT scans in order to verify the incidence of AKI observed.

## Methods

The study cohorts were derived from a large-scale regional quality initiative in the province of British Columbia, Canada, shortly after the publication of the Canadian Association of Radiologists guidelines for the prevention of CIN which involved the use of a protocol aimed at preventing CIN for clinicians ordering CT scans [9]. Specifically, study cohorts were sampled from four hospitals in the region at two time periods: CT scans performed during December 1–12, 2012 before protocol intervention (Period 1) and CT scans performed during October 1–13, 2013, 10 months after protocol intervention (Period 2). Using the health authority’s radiology software, we were able to identify all patients who received CT scans within the participating hospitals during our study periods, as well as details about their scan types. Patient data from the radiology software was then electronically linked with the health authority’s electronic medical records to collect further information including demographics and laboratory information, matched via each patient’s unique identifier code. Collected data were age, sex, type of CT scan, and sCr measurements taken within a 7-day window prior to and after the CT scan. CT scans were categorized into two types: “Contrast” CT scans included scans with administration of IV or IV and oral contrast medium, whereas “Non-Contrast” CT scans included scans in the absence of any contrast medium or oral contrast medium only. Patients were excluded if their CT scans did not involve the head, spine, chest, abdomen or pelvis (i.e. extremities), or if intra-arterial contrast was used, or if a patient received more than one scan within a 7-day period. This study was approved by the Providence Health Care Research Institute ethics board.

The details of the CIN prevention protocol are available in the Appendix. The evaluation of the protocol was not the purpose of the study, but merely provided an opportunity to collect data on convenient samples to explore the incidence of AKI in hospitalized patients who underwent CT scans.

### CIN prevention protocol implementation

Based on current evidence, the CIN prevention protocol was designed to reduce CIN through evidence-based strategies: 1) better surveillance and measurement of serum creatinine pre- and post-contrast scan and 2) mitigate risk by stopping specific medications, and ensuring adequate volume repletion. Radiologists, nephrologists and internists were involved in the development and dissemination of the recommendations. On July 1st, 2013, the protocol was distributed electronically to all staff members within the health authority. At the same time, it became available in paper format throughout every participating hospital within the health authority. There was no pre-printed order, and no electronic decision support tool accompanying the roll out. The use of the protocol was not mandatory in order to order a CT scan, and no mechanism was implemented to track the use of the protocol.

### Outcomes of interest

The primary outcome of interest was AKI as defined as per the KDIGO: an acute change in sCr of ≥26 μmol/L within 7 days post-CT scan [5]. The secondary outcome of interest was the availability of sCr assessment before and after CT scan between Period 1 and Period 2, as a measure of ‘awareness of risk’ by clinicians. SCr availability was grouped into three categories: 1) sCr available before and after CT scan, 2) sCr available either before or after CT scan, and 3) no sCr was available before or after CT scan.

### Statistical methods

Demographic data were summarized by periods and by CT-scan types. Continuous variables were reported in median with interquartile range, categorical variables in frequency with percentages. Two-way ANOVA and logistic regression model were used to examine differences in baseline variables. Logistic regression model was also used to examine the proportion of patients with AKI for the combinations of periods and CT-scan types, adjusting for age, sex and baseline estimated glomerular filtration rate (eGFR) in the model. Specifically, we were interested in the following four comparisons: (i) Contrast CT-scan vs. Non-contrast CT-scan within Period 1, (ii) Contrast CT-scan vs. Non-contrast CT-scan within Period 2, (iii) Period 2 vs. Period 1 among Contrast CT-scans, and (iv) Period 2 vs. Period 1 among Non-contrast CT-scans. To compare the proportions of sCr availability between periods, we fitted a multinomial logistic regression and examined the difference between periods pertaining to the contrast CT-scans only.

A p-value <0.05 was considered as statistically significant. All statistical analyses were performed in SAS, version 9.3 (SAS institute, Cary, NC). Graphical presentation of the data was created in R, version 3.0.1 (R Foundation for Statistical Computing).

## Results

### Derivation of study cohorts and patients’ characteristics at baseline

**Fig. 1:**
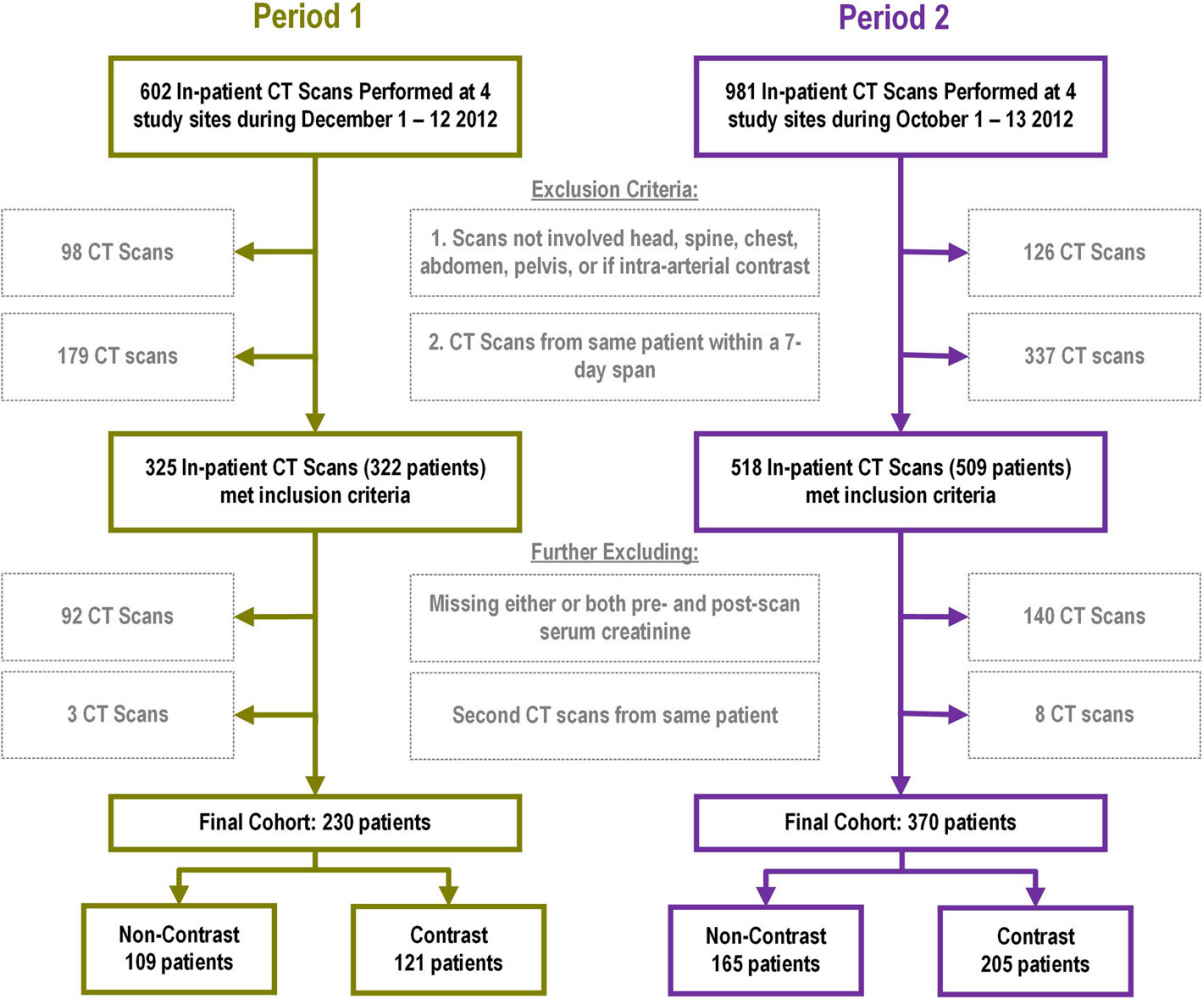
Derivation of study cohorts

Derivation of the study cohorts is depicted in Fig. 1. Slightly more CT-scans were conducted in Period 2; however, the proportion of those excluded for various reasons was similar. Baseline characteristics of the patients are summarized in Table 1. In Period 1, those who underwent CT scans with and without contrast were similar in demographics, but 70 % of patients with contrast CT-scan had an eGFR > 60 mL/min/1.73 m^2^ compared to 57 % of those without contrast (*p* = 0.04). In Period 2, those who received contrast CT scans were younger (median: 65 vs 74 years old, *p* < 0.001) and had a higher eGFR at baseline (median: 74 vs 64 mL/min/1.73 m^2^, *p* < 0.001). There was no difference found between the two periods within either CT-scan types.

**Table 1:** Study cohorts’ characteristics at baseline

	Period 1			Period 2		
	Non-Contrast	Contrast	p-value	Non-Contrast	Contrast	p-value
	CT scan type (*n* = 109)	CT scan type (*n* = 121)		CT scan type (*n* = 165)	CT scan type (*n* = 205)	
Age	70 [59, 81]	68 [54, 77]	0.34	74 [60, 84]	65 [51, 76]	<0.001
Male	57 (52 %)	64 (53 %)	0.93	97 (59 %)	121 (59 %)	0.96
BL eGFR	66 [43, 94]	72 [58, 104]	0.11	64 [37, 87]	74 [58, 96]	<0.001
BL eGFR > 60 mL/min	62 (57 %)	85 (70 %)	0.04	89 (54 %)	147 (73 %)	<0.001

Median [IQR] for continuous variables, frequency (%) for categorical variables

### Incidence of AKI

**Fig. 2:**
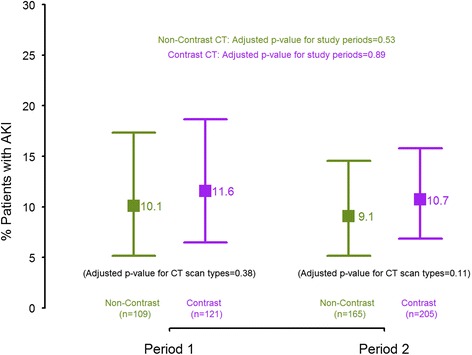
Incidence of AKI by study periods and by CT-scan types

In Period 1, among the 121 patients who received contrast CT scans, the observed incidence of AKI was 11.6 % [95 % C.I.: 6.5 %, 18.7 %] compared to 10.1 % [95 % C.I.: 5.1 %, 17.3 %] in the 109 patients who received non-contrast CT scans. The difference was not statistically significant in both unadjusted and adjusted analyses (p-values ≥ 0.38; see Fig. 2). In Period 2, the incidence of AKI in the 205 patients who received contrast CT scans was 10.7 % [95 % C.I.: 6.8 %, 15.8 %], which was similar to the 165 patients who underwent non-contrast scans with the incidence of AKI observed at 9.1 % [95 % C.I.: 5.2 %, 14.6 %] (p-values ≥ 0.11; see Fig. 2). Among the contrast CT scans, we did not find any difference in Periods 1 and 2 (11.6 % vs. 10.7 %; p-values ≥ 0.81). Similarly, no difference was found between the two periods in those with non-contrast CT scans (10.1 % vs. 9.1 %; p-values ≥ 0.53).

### sCr availability: impact of protocol on ascertainment of renal function

**Fig. 3:**
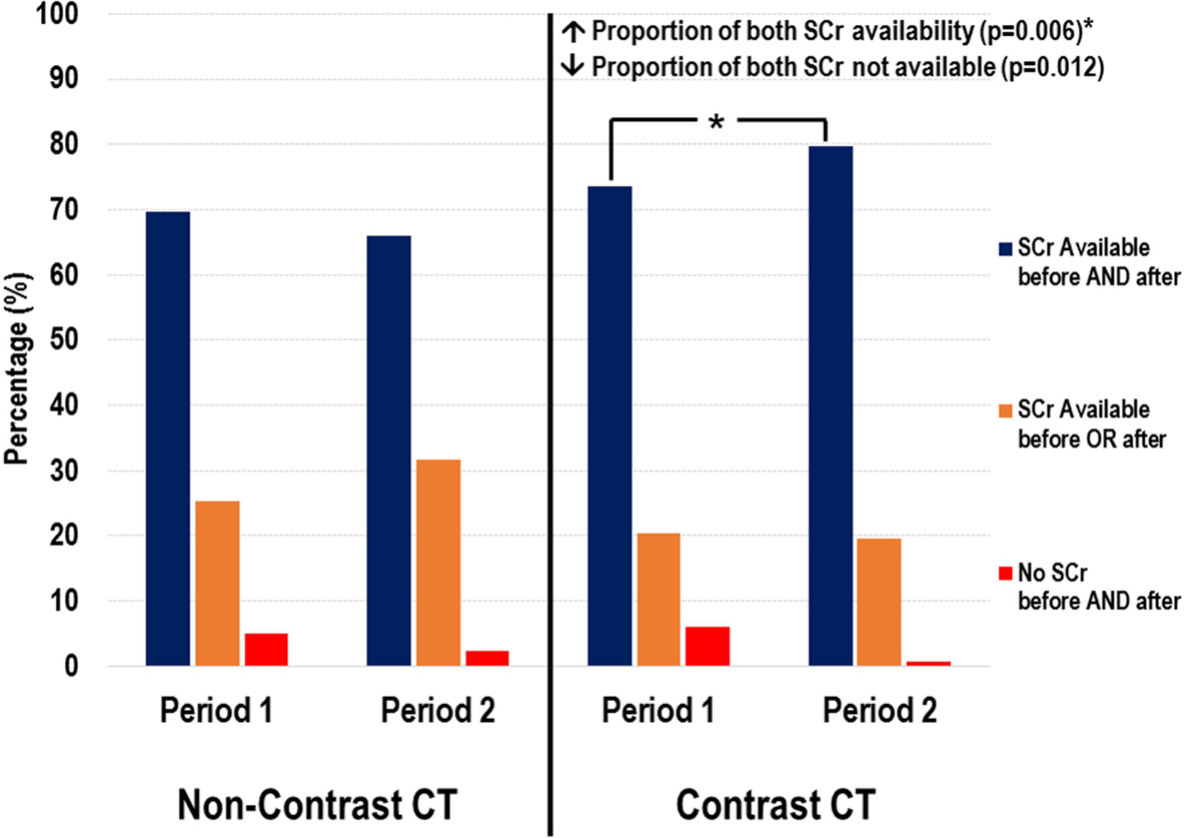
Availability of serum creatinine prior to and post CT scan

Among those who received contrast CT scans, there was a significant improvement in the sCr availability before and after CT scan from 73.6 % [95 % C.I.: 67.6, 80.6] in Period 1 to 79.8 % [95 % C.I.: 75.2, 84.7] in Period 2 (*p* = 0.006, see Fig. 3).

## Discussion

To explore the issues of AKI post-contrast scan in hospitalized patients, our study took advantage of a region-wide quality improvement initiative aimed to mitigate the risk of AKI post-IV contrast CT scan. The incidence of AKI was found to be around 10 %, and similar between patients who did and did not receive IV contrast. In Period 2, the patients with IV contrast were significantly younger and with higher baseline eGFR, potentially reflective of clinician bias of reserving contrast medium for those with the lowest perceived risk of CIN. This phenomenon of “renalism”, or bias against patients with CKD, is described in a study by Chertow et al. [15], where they demonstrated a significant decrease in utilization of coronary angiography among patients with CKD compared to patients with normal renal function, despite similar risk factors and symptomatology. This phenomenon may have been prompted by the quality improvement intervention. Along with the improvement in the monitoring of sCr before and after IV contract scans subsequent to the intervention, our findings suggest that AKI post-contrast may not be related to the administration of contrast medium.

Our results are consistent with recent literature that questioned the etiological mechanisms behind AKI post-administration of IV contrast medium [16–20]. In a retrospective study with over 20,000 CT scans, Davenport and colleagues [16] found that the risk of AKI was similar in both the contrast and the non-contrast group, in those with sCr values >132 μmol/L [16]. Another retrospective study by McDonald *et al.* [20] demonstrated the validity of traditional AKI risk factors in patients who received CT scans, while showing no difference in AKI rates between patients who received IV contrast versus those who did not. These findings echo the McDonald group’s earlier retrospective analysis of over 150,000 CT scans within a 10 year period, showing no difference in AKI risk between the contrast and non-contrast group after propensity adjustment [17]. A recent meta-analysis showed similar results [18]. Collectively, these studies and ours suggest that IV contrast may not be the primary contributor to AKI in patients that receive IV contrast, and referring to those cases as CIN is misleading.

Our study has several strengths. While most studies on the topic involved retrospective data, our data collection was prospective and rigorous in measuring an outcome that is consistent with the current definition of AKI. Given that implementation of this protocol was designed to increase surveillance and detection of CIN with evidence-based measures, one would expect parallel changes in both clinical practice (behavior) and outcome (incidence of AKI). Despite the improved appropriate ordering of sCr pre- and post-contrast scan (i.e. possible ascertainment bias), we still found no change in incidence of AKI. Therefore, the AKI may not be attributable to the contrast medium but may be related to other medical conditions prompting the need for future studies.

Our study has several limitations. This report focused on establishing the benchmark incidence of AKI in patients with and without contrast between two periods. Potentially the lack of difference in rates of AKI between the two time periods was due to poor uptake of the prevention protocol or due to the lack of its intended efficacy; however, the effect of the quality improvement intervention was not addressed, as it was not the purpose of this study. Although it was a multi-centre study, it was limited to one health authority in the province, and its applicability to other places remains to be determined. Nonetheless, the health authority is the most populous in British Columbia, multi-ethnic in composition, and representative of Canadian populations in urban settings [21]. Approximately 20 % of patients who received a contrast scan did not have both a baseline and post-scan sCr, and therefore, we could not determine their incidence of AKI, which may have influenced results. However, the majority of those who did not receive adequate testing were in the lowest-risk group (baseline eGFR > 60 mL/min/1.73 m^2^); therefore, we hypothesize that their inclusion would have only decreased the incidence of AKI post-IV contrast scan even further. We did not perform any multiple pairwise comparisons procedures; however, if we were to apply any correction methods, the conclusions would remain the same as the p-values of our key findings were bigger than the 5 % level of significance. Finally, as with all cohort studies derived from two different time periods, unmeasured epidemiological factors may have impacted our results.

## Conclusion

In conclusion, we are not able to attribute the incidence of AKI in hospitalized patients to the contrast medium use alone given that the incidence of AKI was the same in patients who received CT scans with and without contrast. This may not apply to outpatients where there are fewer confounding factors to the etiology of their AKI. Strategies targeting AKI in general as opposed to specifically contrast-induced AKI in hospitalized patients warrant further study. Future work may focus on better understanding of the determinants of AKI post-contrast scan for developing improved strategies to reduce the incidence of AKI in at-risk individuals undergoing diagnostic scans, albeit lower than previously reported.

## Figures and Tables

**Fig. 4:**
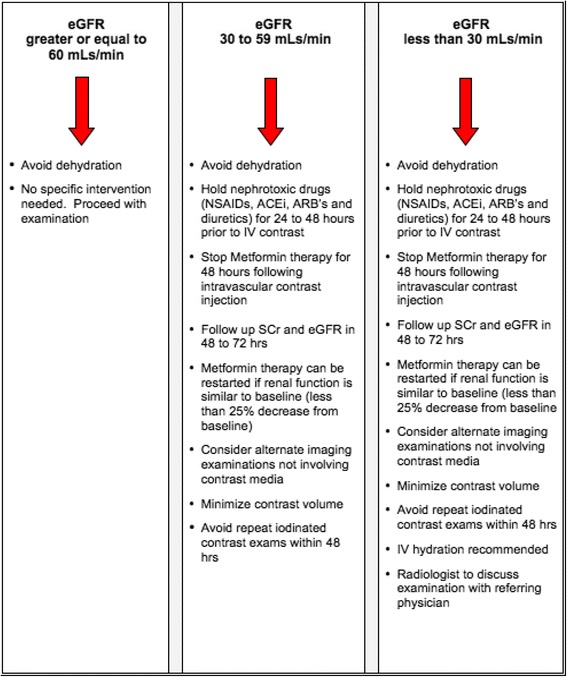
Prevention Protocol to reduce AKI in diagnostic scan involving the use of contrast medium

## Abbreviations

AKI: Acute kidney injury
CIN: Contrast induced nephropathy
IV: Intravenous
eGFR: Estimated glomerular filtration rate
SCr: Serum creatinine
CT scan: Computed tomography scan
